# Determining the stability of minimally displaced lateral humeral condyle fractures in children: ultrasound is better than arthrography

**DOI:** 10.1186/s13018-021-02855-y

**Published:** 2021-11-30

**Authors:** Andreas Rehm, Tamás Kobezda, Elizabeth Ashby

**Affiliations:** Paediatric Division, Department of Paediatric Orthopaedics, Cambridge University Hospitals NHS Trust, Hills Road, Cambridge, CB2 0QQ UK

We read with interest the recent publication by Wu and colleagues [1]. Weiss et al. [2] defined a classification system and treatment for lateral humeral condyle fractures (LHCF) based on fracture displacement and articular congruity: (1) type I displaced < 2 mm: non-operative treatment; (2) type II displaced ≥ 2 mm with intact articular cartilage as seen on arthrogram (all 65 fractures had ≥ 2- < 4 mm displacement): closed reduction and percutaneous pinning (CRPP); (3) type III displaced ≥ 2 mm with articular cartilage not intact (all 93 fractures had ≥ 4 mm displacement on radiographs and were thought to have a disrupted articular cartilage without this having been confirmed by arthrogram or intra-operative findings: open reduction and K-wire fixation (ORIF). The minor/major complication rate was much higher in the type III (23/9) than the type II group (6/1).

Bernthal et al. [3] reported on 141 LCHFs, 76 treated non-operatively, 14 with CRPP and 51 with ORIF. Those treated with CRPP or ORIF had a significantly reduced absolute arc of motion up to 18 weeks after injury compared to non-operative treatment. Nazareth et al. [4] reported significantly reduced Pediatric Outcomes Data Collection Instrument (PODCI) scores for type II/III fractures at 6 and 12 weeks compared to type I fractures.

Li et al. [5, 6] being the same authors as Wu et al. [1] previously reported that the lateral humeral cartilage hinge and fracture stability can be accurately determined with transverse ultrasound (US), based on US examinations performed on children with LHCFs treated between February 2013 and May 2019, without the need for arthrograms.

Li et al. [5] stated that the US technique is simple, safe, inexpensive and the most advanced method for determining fracture stability compared to MRI and arthrography and can effectively avoid inadequate treatment and unnecessary surgery for fractures with 2–4 mm displacement with intact articular cartilage. Of 39 fractures with 2–4 mm displacement 14 had an intact articular cartilage as seen on US and were treated non-operatively without further displacement. This indicates that possibly all of Weiss et al.’s 65 type II fractures could have been treated safely without surgery.

Li et al. [5] recommended the routine use of their US technique. Considering the former information, we agree with Li et al.’s [5] recommendation that US should be introduced as a routine investigation which could change how we manage LHCFs in the future, pushing the frontier of safe non-operative treatment from the below < 2 mm to the ≥ 2- < 4 mm group with intact articular cartilage. This could result in a substantial benefit for this patient group since they would be managed without anaesthetic, would not be exposed to surgical risks and would have a quicker functional recovery [3, 4], with cost savings for overstretched health services.

Despite the same authors [5] having shown that cartilage integrity can be identified accurately purely by using US with the child awake without the need for an arthrogram and it being safe to manage fractures with intact cartilage non-operatively in cast, Wu et al. [1] only described US as a complementary tool with arthrography to assess the integrity of the cartilage hinge. We would like to ask Wu et al. [1] why they started using arthrography in May 2018 under general anaesthetic after having successfully used only US for over 5 years previously and why they performed CRPP on 25 children with ≥ 2- < 4 mm displacement with intact articular cartilage in contrary to them having previously reported that there was neither a need to expose these children to an anaesthetic for an arthrogram nor the need to perform CRPP [5]?

## Data Availability

Not applicable.

## Abbreviations

LHCF: Lateral humeral condyle fractures
CRPP: Closed reduction and percutaneous pinning
ORIF: Open reduction and K-wire fixation
PODCI: Pediatric Outcomes Data Collection Instrument
US: Ultrasound
